# Results after open lunate excision alone or in combination with palmaris longus tendon ball arthroplasty for the treatment of Kienböck’s disease

**DOI:** 10.1186/s13018-023-03953-9

**Published:** 2023-06-30

**Authors:** Fengyu Wang, Li Wang, Li Lv, Wenxu Duan, Yali Xu, Xiaoran Zhang, Xuelin Ma, Zhemin Zhang, Xinzhong Shao

**Affiliations:** 1 Department of Orthopaedic Surgery, The 3rd Hospital of Hebei Medical University, Shijiazhuang, 050051 Hebei People’s Republic of China; 2 Department of Hand Surgery, The 3rd Hospital of Hebei Medical University, Shijiazhuang, 050051 Hebei People’s Republic of China

**Keywords:** Kienböck’s disease, Lunate excision, Tendon ball arthroplasty, Palmaris longus, Comparative outcome study

## Abstract

**Purpose:**

This study aims to compare results after open lunate excision alone and in combination with palmaris longus tendon ball arthroplasty for the treatment of late-staged Kienböck’s disease (KD).

**Methods:**

This is a retrospective study using the prospectively collected data, and patients who had a discharge diagnosis of KD (stage IIIB based on Lichtman staging criteria) and underwent surgical treatment by lunate excision alone or in combination with palmaris longus tendon ball arthroplasty between January 2011 and December 2020 were included in this study. Variables of interest involved demographics, disease condition, operative procedure, and the outcomes evaluated at the last follow-up. Within and between comparisons were performed.

**Results:**

Thirty-five patients underwent lunate excision alone, and 40 patients underwent the combination procedure. At the final follow-up, patients in both groups exhibited significant improvements compared to pre-operation, such as wrist flexion, wrist extension, carpal height ratio, PRWE score, Cooney score, and grip strength (all *P* < 0.05). Compared to the excision group, combination procedure group had significantly longer surgical time (*P* < 0.001), more blood loss (*P* < 0.001) and exhibited better wrist flexion (*P* = 0.001), PRWE score (*P* = 0.001), Cooney score (*P* = 0.0034), and grip strength (*P* = 0.017). The excellent or good rate based on Cooney wrist score was not significantly different (87.5% vs 71.4%, *P* = 0.083).

**Conclusion:**

Lunate excision in combination with palmaris longus tendon ball arthroplasty is a better option than lunate excision alone for the treatment of stage III KD and can be considered as an operative option.

## Introduction

Avascular osteonecrosis of the lunate, also termed as Kienböck’s disease (KD), is a rare disease with a prevalence of less than 5 in 10,000 people [1]. Multiple factors, for example, vascular, anatomic, traumatic, mechanical, and systemic factors, play a role in the disease onset and progression [2, 3]; and in advanced stages (Lichtman III and IV), conditions (i.e., osteonecrosis, fragmentation, and collapse) are irreversible [2], necessarily requiring surgical interventions.

Currently, depending on clinical symptoms and radiologic stages, a variety of surgical modalities are suggested for the treatment of KD, including ulnar lengthening or radial shortening for joint leveling, intercarpal arthrodesis, pedicled or free vascularized bone grafting, interposition arthroplasty using autologous tendon or silicone for the replacement of excised lunate, decompression of the radial metaphyseal core, proximal row carpectomy, and even wrist arthrodesis [4–10]. Although most methods were reported with favorable results, even in the context of a long-term follow-up period [7, 10], the limitations such as limited sample size (< 20 in most studies) and absence of control group should be noted. In addition, the high-demand nature of more sophisticated and costly procedures makes them very difficult to generalize widely to some less equipped institutions and general surgeons, due to the rarity of KD.

On the contrary, there has been a growing focus on low-demand surgical procedures, such as arthroscopic lunate excision and open lunate excision plus tendon ball interposition arthroplasty using palmaris longus, pronator quadratus tendon, or extensor carpi radialis longus, and the results are good and inspiring [11–14]; however, there is still a lack of studies directly comparing surgical modalities. In this study, our objective was to compare radiological and clinical results after open lunate excision alone and in combination with palmaris longus tendon ball arthroplasty for the treatment of late-staged KD.

## Methods

This is a single-center retrospective analysis of the prospectively collected data. The study protocol has been approved by the Ethics Committee of the Third Hospital of Hebei Medical University. All patients provided their informed consent to use their data, with privacy preservation. Patients who had a discharge diagnosis of KD and underwent surgical treatment by lunate excision alone or in combination with palmaris longus tendon ball arthroplasty in our hospital between January 2011 and December 2020 were included in this study, and the relevant data were evaluated. The inclusion criteria were as follows: a definitive diagnosis of KD based on medical history, physical examination, and radiological and MRI findings; stage IIIA or IIIB based on Lichtman staging criteria [5]; and a minimum of 24-month follow-up data. Exclusion criteria were central nervous system diseases or other wrist joint diseases; patients with a history of metabolic diseases, wrist arthritis, wrist fracture/dislocation, or surgery; incomplete or loss of follow-up; or missed data of interest.

## Surgical procedures

### Lunate excision

**Fig. 1:**
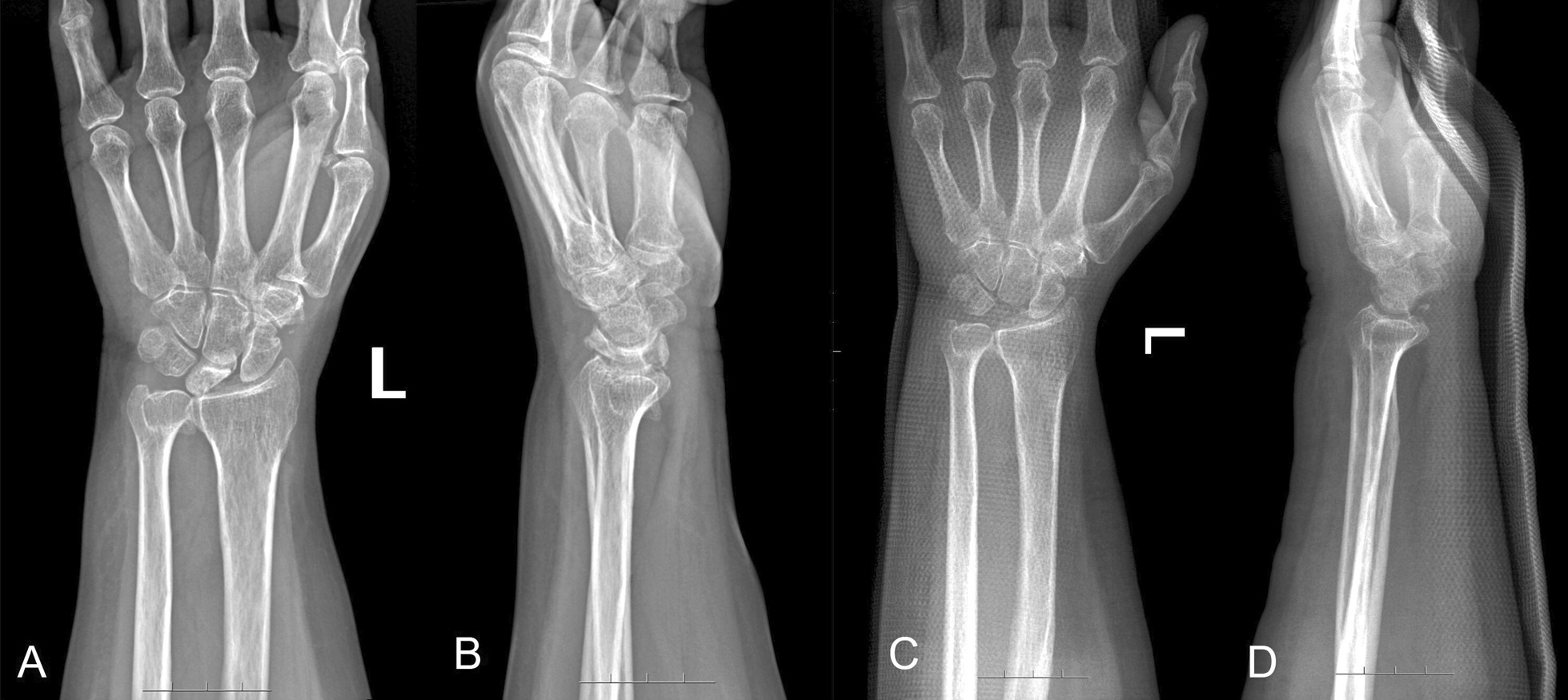
A 46-year-old man who was diagnosed with the left KD (stage IIIb) taken preoperatively (**A** and **B**) and postoperatively (**C** and **D**)

Before surgery, patients were positioned in a supine position with the affected limb abducted and pronated. Brachial plexus anesthesia was administered, and a pneumatic tourniquet was secured to the upper arm to restrict blood flow. A dorsal midline wrist incision, approximately 5 cm in length, was created in an "S" shape. After incising the skin and subcutaneous tissue, the dorsal extensor tendon group was retracted to expose the radial styloid process. This process was utilized as an anchor point to gain access to the lunate and wrist capsule. The necrotic lunate was extracted following excision of the associated ligaments and joint capsule. Subsequent to the confirmation of complete removal of the lunate by fluoroscopy, the wound was irrigated, the tourniquet was removed, and sutures were placed in layers to close the incision. Figure 1A–D presents the radiographs of a 46-year-old man with the left KD (stage IIIb) taken preoperatively (A and B) and postoperatively (C and D).

### Lunate excision in combination with palmaris longus tendon ball arthroplasty

The patients were placed in the supine position with the affected limb abducted and pronated, brachial plexus anesthesia, and a pneumatic tourniquet was placed in the upper arm. An "S"-shaped skin incision, approximately 8 cm in length, was made along the dorsal midline of the wrist. After incision of the skin and subcutaneous tissue, the extensor digitorum tendon was freed and pulled ulnarly. A longitudinal incision was made after exposing the capsule of the lunate joint, and the dorsal ligaments around the lunate were cut. After confirming lunate osteonecrosis, it and its surrounding ligaments were completely removed. Two to three small 1-cm incisions were made on the volar aspect of the wrist, and the palmaris longus tendon was segmentally mobilized. The entire length of the palmaris longus tendon and part of the belly of the muscle were extracted from a small incision at the distal end, and it was sutured into a ball to fill the removed lunate space with 7–0 absorbable sutures and sutured and fixed with the surrounding ligaments. The dorsal ligaments of the radiocarpal and intercarpal joints were repaired, joint capsule and the dorsal extensor retinaculum of the wrist joint were closely sutured, and then, tourniquet is release, followed by thoroughly stopping bleeding, and the wound was sutured layer by layer.

**Fig. 2:**
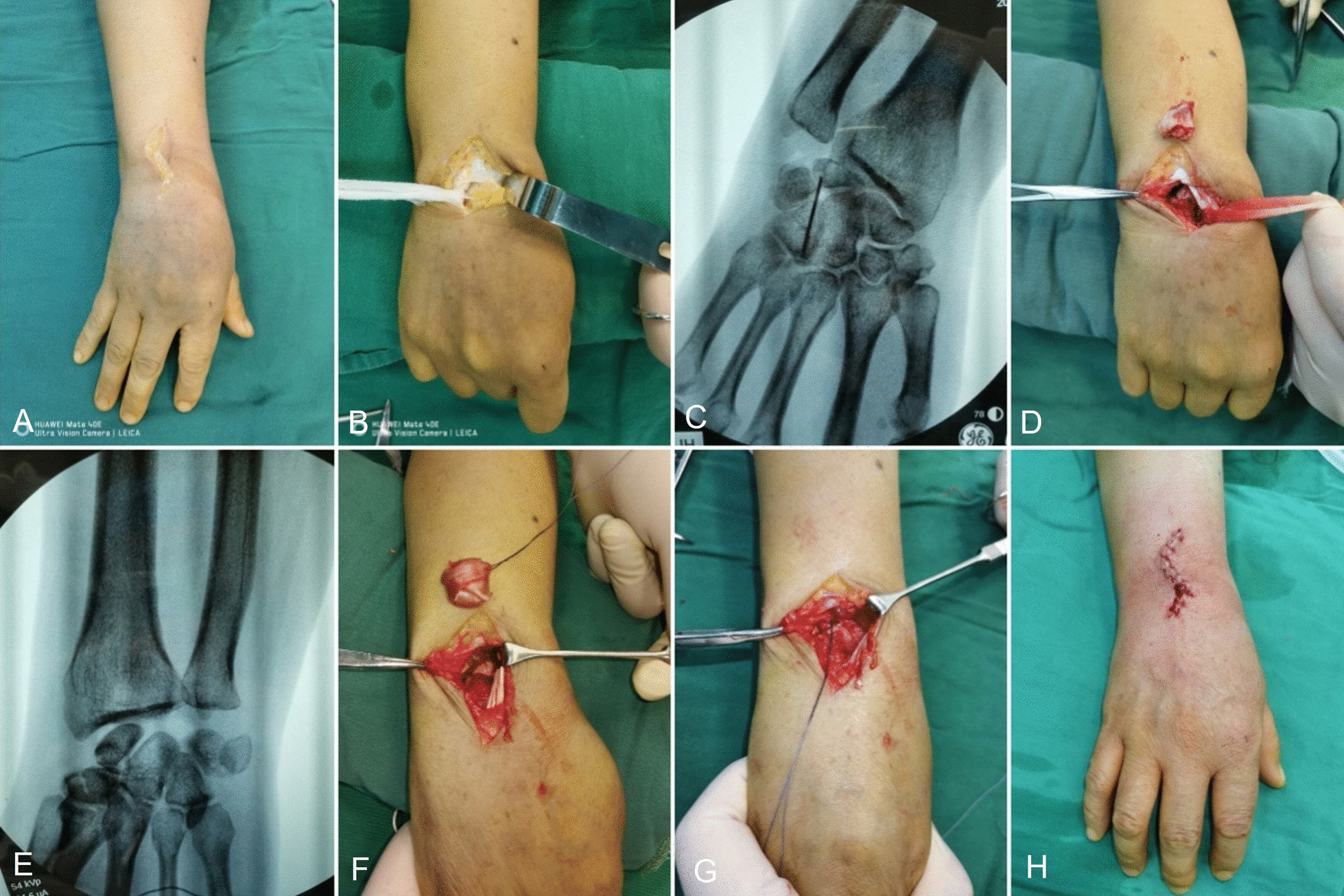
An S-shaped incision was made in the middle of the dorsal wrist (**A**); the extensor tendon was retracted to expose the dorsal wrist joint capsule (**B**); X-ray confirmed the position of the necrotic lunate (**C**); the necrotic lunate was removed, and the defect space was left (**D**); X-ray confirmed that the necrotic lunate was completely removed (**E**); the palmaris longus tendon ball was sutured into a ball (**F**); the palmaris longus tendon ball was placed in the defect space after lunate resection (**G**); and the joint capsule was repaired, and the incision was closed (**H**)

**Fig. 3:**
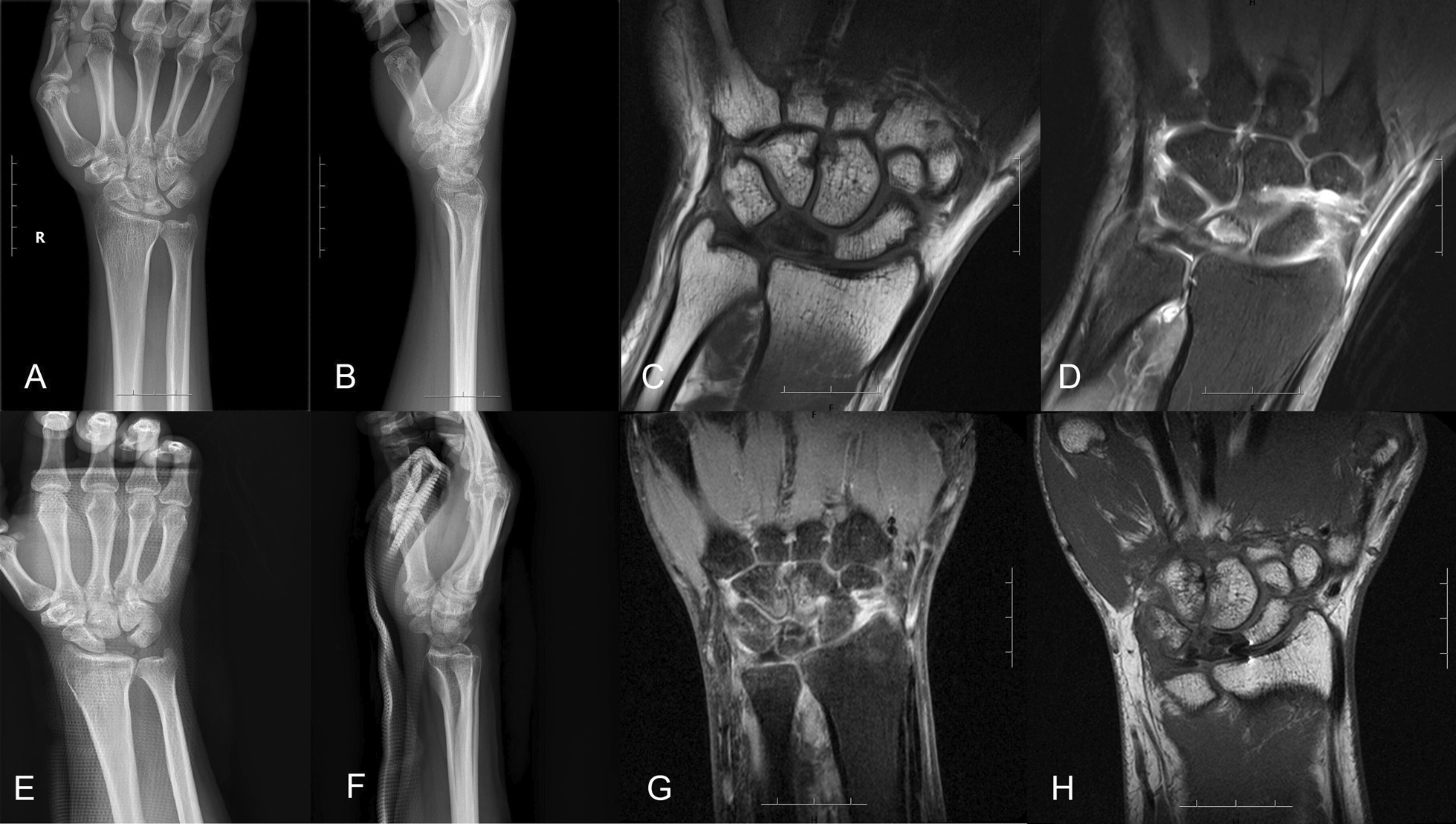
A typical case of stage IIIb of KD in a 40-year-old man in his right wrist, who had a disease course of 6 months, underwent the removal of necrotic lunate and palmaris longus tendon ball arthroplasty. The preoperative radiographs (**A** and **B**) and MRI findings (**C** and **D**) showed the KD in stage IIIb, and postoperative radiographs (**E** and **F**) and MRI findings (**G** and **H**) showed palmaris longus tendon ball interposition in place of removed necrotic lunate

All operations were carried out by the same team headed by the senior author (X Shao). Figure 2A–H presents the detailed steps of the combination procedure. Figure 3A–H presents a typical case of stage IIIb of KD in a 40-year-old man in his right wrist.

### Postoperative management

At the end of the operation, intraoperative blood loss was measured based on the soaked gauze weight and the aspirated fluids. Postoperatively, the same regimen was applied in both groups. After the operation, the patients in both groups were placed in supine position, and the injured limbs were elevated to promote blood circulation and reduce edema. Plaster immobilization in neutral position was used to avoid adhesion of surrounding tissues due to bleeding and to affect recovery of wrist joint function. The stitches were removed approximately 12 days after surgery, and then, the plaster was removed and replaced with functional splinting for the next 2 weeks. Patients were encouraged to perform wrist flexion and extension exercises. Nonsteroidal analgesics were administered as needed.

### Follow-up and outcome evaluation

Routine outpatient follow-up was performed 1, 3, 6, and 12 months after surgery and yearly thereafter. At each visit, wrist anteroposterior and lateral radiographs were taken, patients’ subjective outcomes and objective physical examination findings were evaluated. Carpal height ratio (CHR), ratio of carpal bone height to third metacarpal bone length on anteroposterior radiographs, was measured. Patients were asked to complete the Patient-Rated Wrist Evaluation (PRWE) scale, and the score was calculated to quantify the upper limb function [15]. PRWE is a well-validated wrist-specific questionnaire for evaluating subjective outcomes from pain and function, comprises a total of 15 items (5 items related to pain, 6 related to special activities, and 4 related to daily activities), and the score ranges from 0 to 100, with a lower score indicating a better result [16]. The Cooney wrist score was used to evaluate wrist function, which included four parts (pain symptoms, functional status, grip strength, and range of motion of the wrist), and the score ranged from 0 to 100, with a higher core indicating better recovery of wrist recovery [17]. Based on the calculated score, the outcome was classified as excellent, 90–100 points; good, 80–89; fair, 65–79; and poor, 0–64 points.

### Statistical analysis

For continuous variables, descriptive statistics were expressed as mean and standard deviation (SD); *Shapiro–Wilk* test was used for exploration of normal status, based on which independent Student’s *t*-test or *Mann–Whitney U*-test was used for comparisons and paired t-test or nonparametric Wilcoxon signed-rank test for pre- to postoperative changes (i.e., within-group comparison), as appropriate. The categorical variable was expressed as frequency and percentage, and differences were examined using Pearson’s *Chi-square* or *Fisher’s exact* test (where the expected cell count < 5). *P* < 0.05 was set as the level of statistical significance. SPSS 26.0 software was used to perform all analyses.

## Results

A total of 75 patients were finally included in the present study, including 59 men and 16 females, with an average of 29.2 ± 10.2 years (range, 20–55 years). Most of the patients (68/75) were construction workers, farmers, or manual workers, and 21 patients had a history of direct wrist injury of varying degrees. The affected hand was the dominant hand in 70 cases, the left hand in five cases; the right hand in 60 cases and the left in 15 cases. According to Lichtman staging, 21 cases were classified as stage IIIA and 54 as stage IIIB, respectively. The average duration of the complaint was 21.6 months (range 2–67 months). Most of the patients had a history of conservative treatment, such as local blocking injection with medications, acupuncture, oral medications (e.g., nonsteroidal anti-inflammatory drug), and physiotherapy.

Thirty-five patients underwent lunate excision procedure alone, and 40 patients underwent lunate excision in combination with palmaris longus tendon ball arthroplasty. The baseline characteristics of both the groups and the comparisons are presented in Table 1. We can see that there were no significant differences in terms of demographics, duration of complaint from symptom onset to index surgery, disease classification, range of motion (flexion and extension), grip strength, and follow-up period (all *P* > 0.05).

**Table 1:** Comparisons of baseline variables between the excision group and the combination group

Variable	Combination group (*n* = 40)	Excision group (*n* = 35)	*P*
Age (years)	28.4 ± 5.7	30.2 ± 6.6	0.190
Sex (male)	31 (77.5)	28 (80.0)	0.821
Complaint time since symptom onset	20.9 ± 3.7	22.4 ± 3.9	0.285
Lichtman stage			
IIIa	12 (30.0)	9 (25.7)	0.680
IIIb	28 (70.0)	26 (74.3)	
Wrist flexion (°)	33.6 ± 11.3	31.5 ± 13.7	0.333
Wrist extension (°)	34.8 ± 10.3	35.3 ± 13.6	0.689
Radial deviation (°)	20.3 ± 7.5	20.9 ± 7.2	0.512
Ulnar deviation (°)	29.1 ± 8.3	28.4 ± 9.2	0.214
Carpal height ratio	0.49 ± 0.20	0.49 ± 0.15	0.187
PRWE	49.8 ± 22.3	48.2 ± 21.6	0.679
Cooney score	46.6 ± 17.2	48.4 ± 16.4	0.772
Grip strength (% of the contralateral side)	53.7 ± 8.5	56.3 ± 10.3	0.287
Follow-up period	29.4 ± 6.7	30.2 ± 8.6	0.837

*PRWE* Patient-Rated Wrist Evaluation

At the final follow-up, patients in both groups exhibited significant improvements compared to preoperative values, such as wrist flexion (*P* < 0.001), wrist extension (*P *< 0.001), carpal height ratio (*P *= 0.004), PRWE score (*P *< 0.001), Cooney score (*P *< 0.001), and grip strength of the contralateral side (*P *< 0.001). However, in terms of radial deviation and ulnar deviation, there is slight reduction in both groups, and for ulnar deviation, the reduction was statistically significant (*P *= 0.006 for excision group and *P *= 0.012 for combination group) (Tables 1 and 2).

**Table 2:** Comparisons between the excision group and the combination group at the final follow-up visit

Parameters	Combination group (*n* = 40)	Excision group (*n* = 35)	*P*
Surgical duration (min)	80.1 ± 20.5	50.3 ± 10.4	< 0.001
Hospitalization stay (days)	8.5 ± 2.2	7.7 ± 3.1	0.214
Blood loss (ml)	48.3 ± 10.4	30.6 ± 5.2	< 0.001
Wrist flexion (°)	47.3 ± 5.7	39.4 ± 6.6	0.001
Wrist extension (°)	42.5 ± 9.8	43.7 ± 10.6	0.341
Radial deviation (°)	19.2 ± 8.9	18.8 ± 9.2	0.726
Ulnar deviation (°)	27.6 ± 6.3	25.9 ± 5.2	0.103
Carpal height ratio	0.45 ± 0.05	0.41 ± 0.05	0.069
PRWE score	25.3 ± 1.90	31.7 ± 1.01	0.001
Cooney score	72.8 ± 14.3	65.4 ± 15.7	0.034
Grip strength (% of the contralateral side)	77.3 ± 14.7	68.7 ± 13.6	0.017

*PRWE* Patient-Rated Wrist Evaluation

Compared to the excision group, patients who underwent combination procedures had significantly longer surgical time (*P* < 0.001), more blood loss (*P* < 0.001) and exhibited better wrist flexion (*P* = 0.001), PRWE score (*P* = 0.001), Cooney score (*P* = 0.0034), and grip strength (*P* = 0.017) (Table 2). Based on the Cooney wrist score, an excellent result was achieved in 20 patients, good in 5, fair in the 9, and poor in 1 in excision group and 28, 7, 4, and 1 for combination group, respectively. The excellent or good rate was not significantly different (combination group, 87.5% and excision group, 71.4%; *P* = 0.083).

Complications occurred in the four patients, one in excision group who had a superficial surgical site infection resolved by oral antibiotics and three in combination group, with two having reflex sympathetic dystrophy that resolved with physical therapy and one had subluxation of tendon ball that resolved by secondary surgical intervention.

## Discussion

There is no consensus on the treatment of Lichtman stage III of Kienbock 's disease; however, lunate excision and tendon ball arthroplasty is among the preferred choice. In this study, we reviewed the 75 cases of Lichtman stage III KD who underwent lunate excision alone or in combination with palmaris longus tendon ball arthroplasty procedure in the past 10 years. The results showed that the combination group had significantly better clinical outcomes than the only lunate excision group, in terms of wrist flexion, grip strength, PRWE score, and Cooney wrist score, also the trend toward a higher excellent or good rate (87.5% vs 71.4%, *P* = 0.083).

Wrist kinematics and biomechanical studies demonstrated that the lunate plays a crucial role in wrist movement and joint stability [18]. Complete excision of necrotic lunate would cause damage to the dorsal radiocarpal ligament (DRC) and the dorsal intercarpal ligament (DIC), resulting in dysfunction of the proximal row carpal bone and proximal displacement of the capitate and stress concentration on the scaphoid, further accelerating the collapse of the carpal bones [13]. Therefore, reconstruction of the tendons is the first important thing after excision of the lunate. Shimizu et al. [14] attempted to address this issue by using the arthroscopic excision of lunate, and the minimum 2-year follow-up showed significantly improved pain relief and wrist function recovery in their series of 15 cases. In our study, we repaired part of the dorsal part of the wrist ligaments after removing the lunate. However, the stress on the radial side of the carpal bone will continue to exist, thus increasing the risk of occurrence of painful wrist joint and osteoarthritis over a period. Therefore, patients should be informed prior to surgery about this increased risk, and a longer follow-up period may be better for study purposes.

Lunate excision in combination with tendon ball interposition arthroplasty using various tendons as a spacer, e.g., palmaris longus, pronator quadratus tendon, or extensor carpi radialis longus, has gained increasing popularity in recent years [11–14, 18], and in our institution, palmaris longus tendon is generally applied. Compared to lunate excision alone, lunate excision in combination with tendon ball arthroplasty is superior in maintaining the arrangement of the proximal row of carpal bones, thus providing stability of wrist joint during the postoperative period. This can largely explain our observations of better functional outcomes and ROM using the combination procedure. In other studies, the authors increased wrist stabilization using additive measures, such as Kirschner wire stabilization for 6 weeks [19], or external tractor for traction [20]. On the contrary, in our study, plaster immobilization in neutral position was applied for the first 2 weeks and functional splinting for the next 2 weeks, and we believe that this not only ensures the stability of the wrist joint but may be more conductive to earlier functional recovery.

The carpal height ratio is an important parameter in the prognosis of wrist arthropathy, and maintenance of CHR is essential to prevent progressive degenerative changes at the radioscaphoid joint [21]. Tendon ball arthroplasty by inserting the ball as a spacer provides biomechanical support to the carpus, but slightly poorer than the preoperative value (bony structure, lunate). However, CHR value in combination group is still significantly higher than that in excision group, which suggests the increased risk of degeneration of the radioscaphoid joint. Matschashi et al. [22] tried insertion of an iliac bone flap wrapped in palmaris longus, i.e., forming an osseous core, to maintain CHR to prevent osteoarthritis, and in their 12 cases with a mean follow-up of 45.3 months, two had developed osteoarthris and four had a reduced osseous core. In their study, all the 12 cases had excellent results, likely demonstrating that the clinical results may not be, at least not largely, related to CHR.

The shortcomings of the palmaris longus tendon ball interposition procedure should be noted. The implantation of the palmaris longus tendon ball may develop into scar tissue and calcifications to some extent, which thus reduce the ROM of the operated wrist joint. In Mariconda et al.’s study of 26 cases of KD treated with lunate excision plus palmaris longus tendon ball arthroplasty with a median follow-up of 125 months, MRI findings showed extensive calcification in the defect filled by the tendon ball in all patients, and cartilage damage, synovitis, and erosive or edematous changes in the bones [23]. Second, palmaris longus tendon ball arthroplasty can provide stable support in the early period, but unlikely be able to prevent further collapse and deformation of the carpal bones, possibly causing ligament instability and functional loss [24]. Due to the relatively short follow-up period, we did not have similar adverse events which, however, are predicable in the following years.

This study has several limitations. First, the non-randomization design has made it subject to selection bias, and it is likely that patients undergoing the combination procedure themselves had higher outcomes expectations. Second, due to the rarity of KD, we only enrolled 75 patients, and such a limited sample size was unlikely to produce statistical difference for some rare events, including postoperative complications. Third, the single-center design in a tertiary referral center and a teaching hospital could weaken the generalizability of our results. However, restricting cases to Lichtman stage III would compensate for this bias.

In conclusion, lunate excision in combination with palmaris longus tendon ball arthroplasty is a better option than lunate excision alone, when treating Lichtman stage III KD. A longer period of follow-up is warranted to examine clinical outcomes and the degenerative changes of the wrist joint.

## Data Availability

All the data will be available upon motivated request to the corresponding author of the present paper.

## Abbreviations

KD: Kienböck’s disease
CHR: Carpal height ratio
PRWE: Patient-Rated Wrist Evaluation
SD: Standard deviation
